# Use of Ribosome-Inactivating Proteins from *Sambucus* for the Construction of Immunotoxins and Conjugates for Cancer Therapy

**DOI:** 10.3390/toxins3050420

**Published:** 2011-04-29

**Authors:** José M. Ferreras, Lucía Citores, Rosario Iglesias, Pilar Jiménez, Tomás Girbés

**Affiliations:** 1 Department of Biochemistry, Molecular Biology and Physiology, Faculty of Sciences, University of Valladolid, E-47005 Valladolid, Spain; Email: luciac@bio.uva.es (L.C.); riglesia@bio.uva.es (R.I.); 2 Nutrition and Bromatology, Faculty of Medicine, E-47005 Valladolid, Spain; Email: pilarj@bio.uva.es (P.J.); girbes@bio.uva.es (T.G.)

**Keywords:** ribosome-inactivating proteins, *Sambucus*, immunotoxin, endoglin (CD105), transferrin

## Abstract

The type 2 ribosome-inactivating proteins (RIPs) isolated from some species belonging to the *Sambucus* genus, have the characteristic that although being even more active than ricin inhibiting protein synthesis in cell-free extracts, they lack the high toxicity of ricin and related type 2 RIPs to intact cells and animals. This is due to the fact that after internalization, they follow a different intracellular pathway that does not allow them to reach the cytosolic ribosomes. The lack of toxicity of type 2 RIPs from *Sambucus* make them good candidates as toxic moieties in the construction of immunotoxins and conjugates directed against specific targets. Up to now they have been conjugated with either transferrin or anti-CD105 to target either transferrin receptor- or endoglin-overexpressing cells, respectively.

## 1. Introduction

Ribosome-inactivating proteins (RIPs) are proteins present in some species of plants and bacteria that inhibit catalytically and irreversibly protein synthesis [1,2,3,4,5]. RIPs are *N*-glycosidases (EC 3.2.2.22) that cleave the adenine No. 4324 from the 28S rRNA in the 60S subunit of rat ribosomes (or the equivalent adenine in sensitive ribosomes from other animals) [1,2,3]. This adenine is located in the α-sarcin–ricin loop (SRL) that is involved in the interaction of the ribosome with elongation factor 2 (EF-2) in eukaryotes and elongation factor G (EF-G) in prokaryotes [1,2,3]. RIP-dependent ribosome inactivation arrests protein synthesis by preventing polypeptide chain translocation [6]. Some RIPs also inactivate ribosomes from fungi and certain plants and bacteria. In all these cases, the mechanism of action is the same as that acting on the ribosomes from animals [1,2,3]. Some RIPs also display *N*-glycosidase activity on other adenines from ribosomal RNA [7,8], on viral RNA [9,10] and on genomic DNA [10,11]. Other activities associated with some RIPs are chitinase activity [12], topological activity on DNA [13], HIV integrase inhibitory activity [14], superoxide dismutase activity [15], DNase activity [16], and lipase activity [17,18].

No precise biological role has yet been assigned to RIPs, although it has been postulated that they could have a role in the defense of plants against predators, fungi, and viruses [1,2,3] and that some RIPs could play a role in plant senescence [19].

Plant RIPs have been classified into type 1 RIPs and type 2 RIPs [1,2,3,20]. Type 1 RIPs (such as saporin) consist of a single polypeptide chain that displays the *N*-glycosidase activity, whereas type 2 RIPs (such as ricin) contain two different polypeptide chains linked by a disulphide bridge: An A chain (the active chain, with *N*-glycosidase activity) and a B chain (the binding chain which is able to bind to sugar-containing cell surface receptors). Type 2 RIPs can be heterodimeric or tetrameric. Tetrameric type 2 RIPs (such as *Ricinus communis* agglutinin) are four-chain proteins, consisting of two dimers of the type A-B linked also by a disulphide bridge [2,4,21,22,23]. The B chain allows rapid internalization of the type 2 RIP into the eukaryotic cell, translocation of the A chain into the cytosol, and inactivation of the ribosomes, and for this reason current type 2 RIPs are very toxic proteins. However, a number of nontoxic type 2 RIPs were found in some species from the genus *Sambucus* [24,25,26,27,28,29,30,31,32].

Research on RIPs is expanding because of the interest in their application in human therapy; in particular, cancer, AIDS, and autoimmune diseases [2,3]. The goal of the present review is to comment on the use of ribosome-inactivating proteins from *Sambucus* in the construction of immunotoxins and other conjugates for cancer therapy.

## 2. Ribosome Inactivating Proteins from *Sambucus*

*Sambucus* species have a complex mixture of diverse types of RIPs and related lectins (Table 1). The presence of RIPs and lectins has been studied mainly in *Sambucus ebulus* L. (dwarf elder), *Sambucus nigra* L. (European elder), *Sambucus sieboldiana* Blume ex Graebn. (Japanese elder), and *Sambucus racemosa* L. (red elder). To better classify all the proteins found to date in *Sambucus*, we can divide them into three general groups, type 1 RIPs, type 2 RIPs, and pure homolectins, based on their structure and biological activity.

**Table 1:** Ribosome-inactivating proteins (RIPs) and lectins from *Sambucus* species.

Proteins	Species	Tissues	References
**Type 1 RIPs**			
Ebulitins α, β and γ	*S. ebulus*	Leaves	[33]
Nigritins f1 and f2	*S. nigra*	Fruits	[34]
**Heterodimeric type 2 RIPs**			
Ebulin l	*S. ebulus*	Leaves	[25]
Ebulin f	*S. ebulus*	Fruits	[30]
Ebulins r1 and r2	*S. ebulus*	Rhizome	[29]
Nigrin b, basic nigrin b, SNA I’, SNLRPs	*S. nigra*	Bark	[24,31,35,36]
Nigrins l1 and l2	*S. nigra*	Leaves	[37,38]
Nigrin f	*S. nigra*	Fruits	[26,28]
Nigrin s	*S. nigra*	Seeds	[27]
Sieboldin b	*S. sieboldiana*	Bark	[32]
Basic racemosin b	*S. racemosa*	Bark	[39]
**Tetrameric type 2 RIPs**			
SEA	*S. ebulus*	Rhizome	[40]
SNA I	*S. nigra*	Bark	[41]
SNAIf	*S. nigra*	Fruits	[42]
SNAflu-I	*S. nigra*	Flowers	[43]
SSA	*S. sieboldiana*	Bark	[44]
SRA	*S. racemosa*	Bark	[39]
**Monomeric lectins**			
SELlm	*S. ebulus*	Leaves	[45]
SEA II	*S. ebulus*	Rhizome	[29]
SNA II	*S. nigra*	Bark	[46]
SNAlm and SNAIVl	*S. nigra*	Leaves	[37,38]
SNA IV	*S. nigra*	Fruits	[47]
SNA III	*S. nigra*	Seeds	[48]
SSA-b-3 and SSA-b-4	*S. sieboldiana*	Bark	[49]
SRAbm	*S. racemosa*	Bark	[39]
**Homodimeric lectins**			
SELld	*S. ebulus*	Leaves	[50]
SELfd	*S. ebulus*	Fruits	[30]
SNAld	*S. nigra*	Leaves	[37,38]

*Sambucus ebulus* L., *Sambucus nigra* L., *Sambucus sieboldiana* Blume ex Graebn and *Sambucus racemosa* L. have been shown to contain type 1 RIPs, heterodimeric type 2 RIPs (one A chain and one B chain), tetrameric type 2 RIPs (two A chains and two B chains), and monomeric and homodimeric pure lectins (one or two B chains respectively).

Type 1 RIPs consist of a single polypeptide chain that displays enzymic activity. They have been found in *S. ebulus* leaves (ebulitins α, β, and γ) [33] and *S. nigra* fruits (nigritins f1 and f2) [34].

Type 2 RIPs can be heterodimeric or tetrameric (Table 1). Heterodimeric type 2 RIPs derive from a single precursor comprising a signal peptide and two different domains separated by a linker sequence [32,51,52]. After posttranslational processing, the *N*-terminal region of the precursor yields the A chain with *N*-glycosidase activity whereas the *C*-terminal region is converted into the carbohydrate-binding B chain. Upon expression both chains remain linked by a disulphide bridge. Heterodimeric type 2 RIPs have been found in several parts of *S. ebulus*, *S. nigra*, *S. sieboldiana*, and *S. racemosa* (Table 1). *S. ebulus* contains heterodimeric type 2 RIPs in the leaves (ebulin l) [25], rhizome [29], and fruits [30]. *S. nigra* contains heterodimeric type 2 RIPs in all parts of the plant studied: Bark (e.g., nigrin b) [24,31,35,36], leaves [37], fruits [26,28] and seeds [27]. The bark of *S. sieboldiana* and *S. racemosa* also contain heterodimeric type 2 RIPs [32,39].

Similarly, genes encoding tetrameric type 2 RIPs produce polypetides that have a signal peptide at the *N*-terminus followed by an amino acid sequence containing the A chain, the linker peptide, and the B chain. These polypeptides are processed and, upon proteolytic removal of the linker peptide, produce a heterodimer that contains an A chain and a B chain linked by a disulphide bridge. The union of two heterodimers by another disulphide bond between the two B chains yields the tetrameric protein. Tetrameric type 2 RIPs are present in the bark of the perennial trunk of *S. nigra* [41], *S. sieboldiana* [44] and *S. racemosa* [39], the fruits of *S. nigra* [42], the perennial root system of *S. ebulus* [40] and the flowers of *S. nigra *[43]. The lectin subunit of all the tetrameric type 2 RIPs from *Sambucus* specifically binds to the Neu5Ac(α-2,6)Gal/GalNac sequence [44,53]. This makes these lectins unique and different from other type 2 RIPs either from *Sambucus* or other families.

The third group corresponds to the lectins, which do not show enzymic activity and present only lectin activity. They can be homodimeric (two type B-chains held together by a disulphide bridge) or monomeric (one single type B-chain). The precursors of these lectins display a striking sequence identity with type 2 RIPs in the signal peptide, in the first amino acid residues of the A-chain and in the linker region between the A and B chains of type 2 RIPs. The lectin precursor is converted into the mature protein through a processing mechanism where the signal peptide, a small part of the A chain precursor, the connecting peptide and in some cases few residues of the *N*-terminal amino acid sequence of the B chain are lost [37]. In the homodimeric lectins, a new cysteine appeared which is responsible for the dimerization of the lectin polypeptide chain through an interchain disulphide bridge [50]. However, some mature monomeric lectins are processed from a precursor containing a signal peptide followed only by the mature polypeptide. The structure of these lectins also showed a large homology with that of the B chain of type 2 RIPs [49]. Monomeric lectins are present in the leaves [45] and rhizomes [29] of *S. ebulus*, in the leaves [37], bark [46], fruits [47] and seeds [48] of *S. nigra*, and in the bark of *S. sieboldiana* [49] and *S. racemosa* [39]. The homodimeric lectins are found in leaves [50] and fruits [30] from *S. ebulus* and leaves [37] from *S. nigra*. There is also evidence for the occurrence in *S. nigra* bark and fruits of small lectins consisting of a truncated part of the B chain of the tetrameric type 2 RIP SNAI found in the same tissues [42].

The phylogenetic analysis supports the figure of a common two-chain gene ancestor for all these proteins [37]. The proteins from all the three *Sambucus* species tends to be grouped based on their putative structures rather than species relationship. These facts imply that the ancestral *Sambucus* RIP gene existed as a single gene in the ancestral lineage, and duplications of the type 2 RIP gene occurred prior to the divergence of *Sambucus* species. Therefore, the *Sambucus* proteins evolved from a small number of ancestral genes that have undergone multiple events of gene duplication and excisions. The phylogenetic tree of *Sambucus* type 2 RIPs and lectins show two major clades [37]. One of these clades contains both heterodimeric and tetrameric type 2 RIPs either specific for Neu5Ac(α-2,6)Gal/GalNAc or devoid of carbohydrate-binding activity. The type 2 RIP ancestral gene gave rise to another clade grouping all the Gal/GalNAc-specific proteins which can be subdivided in two groups. One group contains homodimeric lectins, most probably formed by excision of almost the complete A-chain domain, and characterized by the presence of an extra cysteine, responsible for protein dimerization through a disulphide bridge. This cysteine residue differs from that involved in the dimerization of the B chain of the tetrameric lectins. The second cluster grouped heterodimeric type 2 RIPs together with monomeric lectins. These monomeric lectins could be encoded by a truncated type 2 RIP gene which has lost a substantial part, if not all, coding for the A chain and therefore lost its enzymic activity. To date, there is no sequence data available on type 1 RIPs from *Sambucus* because the *N*-terminals of the proteins studied are blocked [33].

## 3. Structure of Type 2 RIPs from *Sambucus*

**Figure 1:**
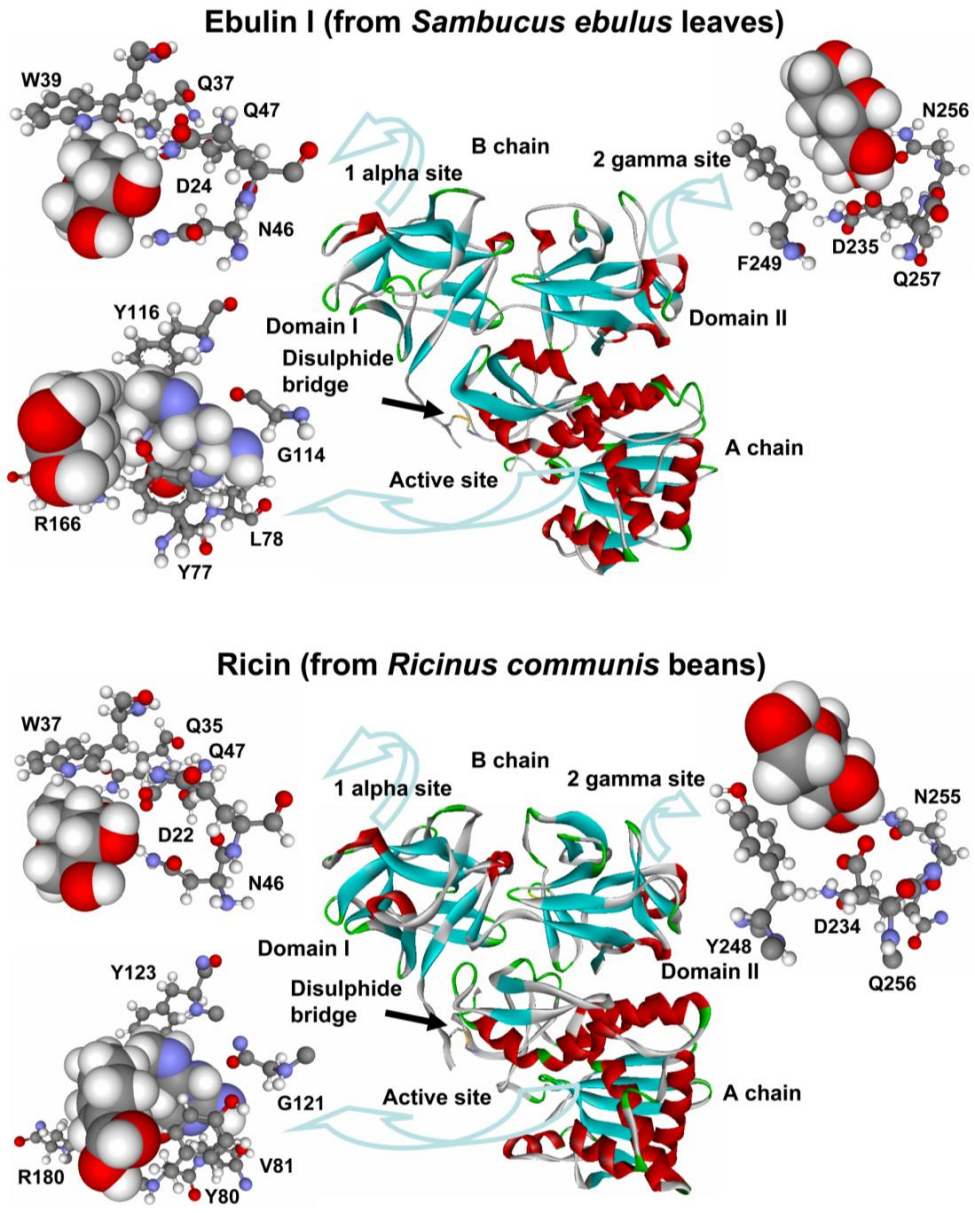
Three-dimensional models of ebulin l and ricin. The disulphide bridge between the A and B chains is indicated. The active sites (balls and sticks) of ebulin l and ricin bound to the substrate analog pteroic acid (CPK) and the sugar-binding sites 1 alpha and 2 gamma (balls and sticks) bound to D-galactose (CPK) are emphasized. The key residues in ebulin l and the corresponding residues of ricin are indicated.

The structure of ebulin l (a heterodimeric type 2 RIP present in *S. ebulus* leaves) has been resolved by X-ray diffraction analysis and it closely resembles that of ricin [52] (Figure 1). In the A chain, ebulin l has roughly the same positioning of key active site residues as ricin, with the exception that the side chain of the Tyr 77 (Tyr 80 in ricin) of ebulin l is rotated out of the binding site pocket. This orientation is similar to that seen in PAP (a type 1 RIP from *Phytolacca americana* L. [2]). Pteroic acid, an A chain substrate analog, binds in a similar way to both active sites (Figure 1). The pterin ring stacks with the side chain of Tyr 77 and forms hydrogen bonds with the backbone of Leu 78 (Val 81 in ricin) and Gly 114 (Gly 121 in ricin). Arg 166 (Arg 180 in ricin) donates a hydrogen bond to N5 of the pterin. The similarity between both active sites is consistent with the fact that both proteins have a similar inhibitory activity of protein synthesis (Table 2).

The overall fold of the ebulin B chain is also very similar to that of the ricin B chain (Figure. 1) and is composed of two beta trefoil domains (I and II) with sugar-binding ability. Ricin binds both galactose and lactose in the subdomains I and II (in the 1 alpha and 2 gamma sites, respectively). Ebulin l binds to galactose and lactose by its 1 alpha site in a nearly identical fashion to ricin (Figure 1) and uses the same sugar-binding residues as ricin: Trp 39 (Trp 37 in ricin), Asp 24 (Asp 22 in ricin), Gln 37 (Gln 35 in ricin), Asn 46 (Asn 46 in ricin), and Gln 47 (Gln 47 in ricin) [52]. Although ricin and ebulin l have very similar 2 gamma binding site geometries, lactose does not bind to the ebulin l 2 gamma site. The mode of galactose binding in the 2 gamma site of ebulin l is somewhat different (Figure 1): The orientation and the positioning of galactose within the binding cleft are shifted as compared with ricin, and galactose bound to ebulin l is located further into the binding cleft than the galactose moiety bound to ricin. This would cause steric interference for any sugar attached to the C1 hydroxyl and this could be reason why lactose does not bind to the 2 gamma site of ebulin l. This altered mode of galactose binding in the 2 gamma site of ebulin l may indicate a weaker binding to complex sugars. In fact, it was found that ebulin l, nigrin b, and the lectins SELlm and SELld have different binding properties to D-galactose containing matrix than ricin [45,52]. Notably, and unlike ricin, the binding to this matrix was dependent on temperature, being maximum in the range of 0–10 °C and abolished at 20 °C [45]. These differences could indicate that the binding to cell surfaces may be altered o diminished for *Sambucus* RIPs and lectins.

## 4. Toxicity and Intracellular Pathway of Type 2 RIPs from *Sambucus*

All the type 2 RIPs from *Sambucus *show a considerable lower cytotoxicity than ricin and the other toxic type 2 RIPs (Table 2). The heterodimeric type 2 RIPs, ebulin f and l, nigrin b and f, and sieboldin b (in contrast to ricin, abrin and volkensin) display very low toxicity to HeLa cells (IC_50_ values higher than 2900 pM) [24,25,32,54,55]. In mice, the LD_50_ of *Sambucus* heterodimeric type 2 RIPs administered by intraperitoneal injection is higher than 1.6 mg/kg body weight while ricin, abrin or volkensin are lethal at concentrations in the range of few microgram/kilogram (Table 2).

**Table 2:** Cytotoxicity of heterodimeric type 2 RIPs from *Sambucus*.

	Rabbit Lysate IC_50_ (pM) ^a^	HeLa Cells IC_50_ (pM)	Mouse LD_50_ (µg/kg)	References
Ricin	100	0.67	3.00	[11,54,56]
Abrin	500	3.70	0.56	[57]
Volkensin	370	0.30	1.38	[20]
Ebulin f	30	17,000.00	>1600.00	[30,38]
Ebulin l	150	64,300.00	2000.00	[25,54,38]
Nigrin b	30	27,600.00	12,000.00	[24,54,38]
Nigrin f	30	2900.00	>1600.00	[26,55,38]
Sieboldin b	15	11,800.00	>1600.00	[32]

The table shows the effects of heterodimeric type 2 RIPs from *Sambucus* on protein synthesis by a cell-free system derived from rabbit reticulocytes lysates and toxicity to intact cells and animals compared to ricin, abrin and volkensin. ^a^ Reduced toxin.

**Figure 2:**
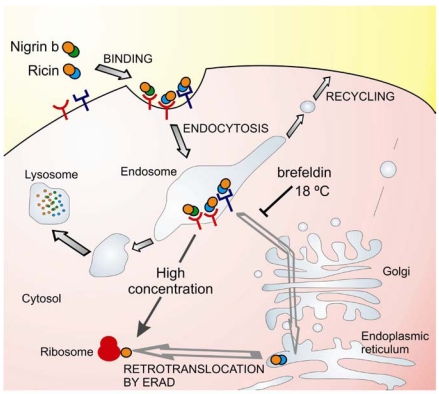
Intracellular trafficking of nigrin b and ricin. Ricin (at pM concentrations) binds to glycoproteins of the plasma membrane and internalize into the cell. Some protein molecules are recycled back to the plasma membrane, others undergo degradation in the lysosomes, and a small number are transported first to the Golgi network and then to the endoplasmic reticulum. In the endoplasmic reticulum, the disulphide bridge is reduced and the A chain translocates to the cytosol by the endoplasmic reticulum-associated degradation (ERAD) pathway. In the cytosol, the A chain inactivates the ribosomes, inhibiting protein synthesis and causing cell death. This pathway is sensitive to low temperature and brefeldin A. Nigrin b (at pM concentrations) can bind to different glycoproteins of the plasma membrane than ricin and internalize into the cell. All the protein molecules are either recycled back to the plasma membrane or transported to lysosomes for degradation. This pathway is not sensitive to low temperature and brefeldin A. However, at much higher extracellular concentration (40,000 folds), the saturation of the endosome with nigrin b can lead to a spontaneous release of nigrin b into the cytosol, causing ribosome inactivation.

Possible explanations for the low toxicity of nigrin b as compared with ricin in HeLa cells have been investigated studying the binding, uptake, intracellular trafficking and processing in cells [55,58,59]. Binding of these RIPs to glycoprotein receptors occurs prior to internalization and intracellular transport and it has been shown that ricin binds to HeLa cells to a greater extent than nigrin b [58,59]. The high toxicity of ricin for mammalian cells is related to its ability to bind and to be transported to the endoplasmic reticulum, and to reach the ribosomes via the endoplasmic reticulum associated degradation pathway (ERAD) [60,61]. Ricin toxicity is sensitive to brefeldin A and to low temperature [62]. In contrast to ricin, nigrin b and ebulin l follow a pathway that is insensitive to brefeldin A and to temperatures below 37 °C indicating that transport from endosomes to the Golgi complex is not required for nigrin b and ebulin l A-chain translocation [55,58]. In fact, nigrin b was found to enter cells like ricin, but was more rapidly and extensively degraded and when excreted by HeLa cells the nigrin b-derived material was completely inactive [59]. In an attempt to explain the lack of cellular toxicity of nigrin b as compared with ricin, we formulated the hypothesis that the internalization of ricin and nigrin b might involve different receptors and therefore they could follow different intracellular pathways (Figure 2). Some of the receptors would carry nigrin b or ricin as a receptor—RIP complex that is either recycled back to the plasma membrane or transported to lysosomes for degradation. This would not be a productive pathway for the internalization of type 2 RIPs. Other receptors would carry ricin and the related highly toxic type 2 RIPs (e.g., viscumin, abrin, modeccin, and volkensin, but not nigrin b) through the endosomal pathway and at some point it diverges to the trans-Golgi network. From there, the proteins are retrogradely transported to the endoplasmic reticulum, where temperature-dependent translocation of the A chain to the cytosol occurs [62]. The cellular uptake of ricin by a combination of both putative internalization pathways could account for the results reported previously [54,59] namely, high cellular toxicity, and substantial degradation. In contrast, cell protein synthesis inhibition by nigrin b seems to be a consequence of the spontaneous translocation of nigrin b from the endosome when the extracellular concentration of RIP is high.

## 5. Use of RIPs and Lectins from *Sambucus* for the Construction of Conjugates for Cancer Therapy

Interest in RIPs has increased in recent years because of their use as the toxic moieties of conjugates for targeting of the experimental therapies. Conjugates are proteins that contain a toxin conjugated to an antibody or ligand by genetic fusion or by chemical ligation; the targeting protein or ligand provides the ability to target specifically the conjugated toxic moiety to the cells. In the case of monoclonal antibodies, the conjugates are called immunotoxins (ITs). When the targeting moiety is a cytokine, growth factor, transferrin or peptide hormone, the molecule is usually referred to as a chimeric toxin. Many protein toxins have been used to make ITs or chimeric toxins [63,64,65]. The most common toxic moieties used are derived from either bacteria (e.g., *Pseudomonas* exotoxin (PE) or diphtheria toxin (DT)), or plants (e.g., ricin or abrin) [66,67,68]. Nearly all protein toxins kill cells by enzymatically inhibiting protein synthesis. For example, PE and DT inactivate elongation factor 2 (EF-2) and ricin and abrin inactivate the EF-2 binding site on the 28S ribosomal subunit. These toxin moieties require internalization and translocation to the cytosol to achieve the cytotoxic effect.

Conjugates, composed of protein toxins connected to cell binding ligands, have been developed over several decades and encouraging clinical trials have been carried out to target different malignancies [63,65,69,70]. Among the most active are those targeted to tumors, specifically those directed against hematological tumors [63,71]. However, obstacles to successful treatment of solid tumors include poor penetration into tumor masses, toxicity and the immune response to the toxin component [2,72,73]. Therefore, exploring different classes of toxins to create new ITs may constitute an alternative strategy to improve the treatment of cancer.

The RIPs from *Sambucus*, nigrin b and ebulin l are 10^3^–10^5^ times less toxic in cultured cells and mice than ricin (Table 2). In contrast, the anti-ribosomal molecular actions of nigrin b, ebulin l and ricin are roughly the same (Table 2). Ricin has been the most used RIP in the construction of conjugates and immunotoxins for targeting cancer cells [63,74,75,76]. Immunotoxins with the ricin holoenzyme, its A chain, and the ricin holoenzyme with the sugar-binding domains blocked to reduce its unspecific activity, have been constructed [77,78]. The lack of toxicity of type 2 RIPs from *Sambucus* make them excellent candidates as toxic moieties in the construction of immunotoxins and conjugates directed against specific targets.

### 5.1. Transferrin-Nigrin b/Ebulin l Conjugates

Transferrin (Tf) is a monomeric glycoprotein involved in the transport of iron throughout the body. The transferrin receptor (TfR) can be targeted by direct interaction with conjugates of its ligand Tf or by monoclonal antibodies specific for the TfR. The TfR is ubiquitously expressed on normal cells and expression is increased on cells with a high proliferation rate or on cells that require large amounts of iron. Expression of the TfR is significantly upregulated in a variety of malignant cells and in many cases, increased expression correlates with tumor stage and is associated with poor prognosis [79,80]. Investigations targeting the TfR thus provide an important approach to hamper cell proliferation due to: (1) the high TfR expression on the surface of most malignant cells, (2) high efficiency of TfR internalization and (3) fast recycling of the receptor once internalized. Tf has been conjugated to cytotoxic agents (e.g., diphtheria toxin, ricin and gelonin) to study their ability to target these toxins to tumor cells, while reducing their non-specific toxic effects on non-tumor cells [81]. It has been shown that the expression of transferrin receptors by human cancer cells is directly correlated with the anti-tumor effectiveness of anti-transferrin receptor conjugates [82,83,84]. Transferrin has also been used for anticancer drug delivery in cancer chemotherapy [85]. 

Based on these data, nigrin b and ebulin l were conjugated to human transferrin to study their potential suitability for the construction of conjugates for cancer therapy [86]. Both conjugates (Tf-nigrin b, Tf-ebulin l) were prepared using SPDP (*N*-succinimidyl-3-(2-pyridyldithio)propionate) as a linker. Conjugation of nigrin b and ebulin l to Tf did not affect largely their translational inhibitory activities on a cell free protein synthesis carried in rabbit reticulocyte lysates with IC_50_ values of 5 and 20 ng/mL for Tf-nigrin b and Tf-ebulin l, respectively. Since free transferrin does not inhibit protein synthesis even at high concentrations, the inhibitory activity of conjugates was dependent on the presence of the conjugated RIP.

To assess the ability of the conjugates to inhibit cell protein synthesis, their effect on HeLa cells was investigated. Incubation of cells with either Tf-ebulin l or Tf-nigrin b led to the inhibition of cell protein synthesis, with an IC_50_ of 100 ng/mL. In contrast, both free forms of ebulin l and nigrin b showed much higher IC_50_ values, close to 4000 and 1800 ng/mL, respectively. To confirm specificity of activity, excess Tf was used in competition experiments to block the human transferrin receptor and reduce the Tf-conjugate activity. On the other hand, it is noteworthy that the small but consistent and repetitive difference in inhibitory action between nigrin b and ebulin l in the rabbit reticulocyte lysates (Table 2) disappeared upon conjugation with Tf, indicating that the inhibitory effect of both conjugates was dependent only on the recognition, binding and internalization of Tf-receptors. The ricin cytotoxicity for HeLa cells under the same conditions as those used in this study for ebulin l and nigrin b is much higher (IC_50_ 0.06 ng/mL) [54]. The IC_50_ values for both conjugates expressed as RIP content would be 300 pM, which lies in the range and is even lower than the values reported for other Tf-RIPs and anti-Tf receptor-toxin conjugates used in therapy [82,84,87]. Therefore, conjugation of nigrin b or ebulin l to human transferrin not only fails to affect the intrinsic translational inhibitory activity of these RIPs but also increases their toxicity to target cells through the Tf receptor. The differences in cytoxicity between free and conjugated non-toxic type 2 RIPs are crucial for the suitability of these RIPs as the intracellular toxic moieties of immunotoxins and conjugates. In all these cases, what seems mandatory is the intracellular traffic and fate of the Tf receptor upon interaction with Tf, whether alone or linked to a protein effector. This is in agreement with data reported on the effects of the anti-Tf receptor immunotoxins, which suggests that the efficacy of immunotoxins is more determined by the rate of internalization (increasing their intracellular concentration) and their fate (routing them to a more productive compartment) than by receptor numbers at the plasma membrane [88,89].

### 5.2. Anti-Endoglin-Nigrin b/Ebulin l Immunotoxins

Nigrin b and ebulin l have been used to also construct immunotoxins containing anti-human endoglin to target tumor neovasculature which nourishes tumor cells [90,91,92]. Tumor progression is characterized by the formation of a neovasculature, which supplies tumor cells with oxygen and nutrients. The formation of new blood vessels (angiogenesis) is necessary for the growth and metastatic spread of solid tumor [93,94,95,96]. The growth of cancer cells inside the solid tumor induces an increase of the interstitial pressure, which forms a barrier to transcapillary transport. This barrier is an obstacle in tumour treatment, as it results in inefficient uptake of therapeutic agents, thus leading to the reduced effectiveness of conventional chemotherapy drugs [97,98]. The anti-angiogenic therapy represents one of the most promising modalities for cancer treatment, as an alternative adjuvant to traditional anti-cancer therapies [93,94]. The goal of the anti-angiogenic approach is to deliver an effector or cytotoxic agent specifically to the vasculature of a solid tumor to eliminate blood supply to the tumor. Such an anti-angiogenic approach enables the reduction of the concentration of the conventional anti-cancer drugs with potential harmful side effects and overcomes several limitations such as the acquired resistance to certain chemotherapy drugs and the need to target a heterogeneous malignant cell population [93,99].

A relatively large number of anti-angiogenic compounds have been found and some of them are already in clinical trials [96,100]. Several antibodies with anti-angiogenic activity are currently under active clinical investigation for cancer treatment, and recently, the humanized anti-VEGF monoclonal antibody bevacizumab, has received approval by the Food and Drug Administration (USA) for selected clinical indications [99,101].

Another anti-angiogenic approach is the targeting to the tumor neovasculature with immunoconjugates. Immunoconjugates contain an antibody raised against a plasma membrane surface antigen and a cytotoxic agent, for example a radioactive isotope [102] or a toxin, for instance plant ribosome-inactivating proteins (RIPs) [63]. 

Over the years great effort has been made to find specific markers for the angiogenic endothelial cells that can be used by vascular targeting agents. One biomarker of proliferation-dependent pathologies is CD105 (endoglin), a TGF-β coreceptor highly expressed in proliferating endothelial cells of the new vasculature and upregulated by hypoxia [99,103,104]. Several studies have suggested that endoglin is a specific marker of neovascularization in various cancer types [105,106,107,108,109,110,111]. A clear implication of endoglin in cancer is that the plasma level of soluble endoglin appears to correlate with metastasis in patients with breast cancer [112]. In addition, endoglin is expressed minimally in benign tissues but strongly in malignant tumors [104,113,114]. These findings support the role of endoglin as an optimal marker of proliferation of endothelial cells and their usefulness for therapeutic anti-angiogenic approaches in human cancer.

The anti-tumor potential of RIPs has been demonstrated in clinical trials with inmunotoxins [63]. In fact, some ricin A-chain immunotoxins targeting human CD105 are active in the prevention of the growth of human tumors grafted in nude mice [115,116].

The *Sambucus* RIPs, ebulin l and nigrin b have been used as the toxic part of two immunotoxins containing the mouse monoclonal antibody 44G4 raised against human CD105 as a carrier molecule [91,92]. Both immunotoxins were formed by covalent linking of the RIP and endoglin with SPDP and were purified by chromatography on Superdex 200. In contrast to some immunotoxins made with blocked ricin, the analysis of the anti-ribosomal effects in a cell-free translation system indicated that conjugation did not affect the activity of ebulin l or nigrin b (Table 3). The IC_50_ for both immunotoxins were 88 pM and 150 pM for 44G4-nigrin b and 44G4-ebulin l, respectively. 

To assess the cytotoxicity of the immunotoxins, their effects on human CD105+ cells such as the mouse fibroblast L929 cells transfected with the short form of human CD105 (L929(S), was investigated (Table 3). Both 44G4-ebulin l and 44G4-nigrin b displayed cytotoxicity with picomolar IC_50_ values on human CD105+ cells. Nigrin b immunotoxin kills specifically L929(S) cells with an IC_50_ value of 600 pM while nigrin b alone kills at 240,000 pM. The immunotoxin was completely ineffective on parental L929 cells. 44G4 monoclonal antibody, even linked to nigrin b, does not recognize the murine CD105 present in both the parental and the transfected L929 cells [117]. A strong cytotoxic effect was observed on the viability of HUVEC (human umbilical vascular endothelial cells), which express high levels of endoglin during their proliferation phase in culture [112]. Immunofluorescence analysis indicated that 44G4-nigrin b accumulated in a perinuclear region [91], demostrating that human CD105 is internalized in transfected cells and allowed to promote the intracellular action of nigrin b either through the inhibition of protein synthesis or apoptotic mechanisms as reported for ricin in some cell lines and *in vitro* [2] and for nigrin b *in vivo* [56].

44G4-ebulin l was also very effective on human CD105+ cells like the mouse fibroblasts L929 cells transfected with the short form of human CD105 (L929(S) cells) and rat myoblasts L6E9 transfected with the long form of human CD105 (L6E9(L) cells) (Table 3). The effect of the 44G4-ebulin l immunotoxin on transfected L929(S) cells was observed with an IC_50_ value of 310 pM. In contrast, cells lacking human CD105 were 2–2.5 logs less sensitive to the immunotoxin [92].

From these data we can conclude that with CD105 being considered as a potential target for the anti-vascular therapy of tumors, nigrin b and ebulin l can be used to construct potent and antigen-specific immunotoxins for anticancer therapy.

**Table 3:** Cytotoxicity of anti-human endoglin immunotoxins containing either nigrin b or ebulin l on endoglin-expressing cells.

	IC_50_ (pM)					
	Nigrin b	Ebulin l	44G4 − Ng b	44G4 − Eb l	44G4 + Ngb	44G4
**Protein synthesis**						
Lysate	25	150	88	150	-	-
L929 (48 h)	10,000	-	>10,000	-	-	-
L929(S) (24 h)	100,000	-	1560	-	-	>10,000
L929(S) (48 h)	14,500	-	188	-	-	>10,000
**Cell viability**						
L929 (48 h)	200,000	>10,000	170,000	10,000	-	-
L929(S) (48 h)	240,000	>10,000	600	310	>10,000	-
HUVEC (48 h)	200,000	-	6400	-	-	>10,000
L6E9 (48 h)	-	>10,000	-	>10,000	-	-
L6E9(L) (48 h)	-	>10,000	-	4000	>10,000	-

The table shows the effect of anti-human endoglin immunotoxins containing either nigrin b (44G4-Ng b) or ebulin l (44G4-Eb l) on a cell-free system (rabbit reticulocyte lysates), human endoglin-expressing cells (L929-S, L6E9-L, HUVEC), and human endoglin-non expressing cells (L929, L6E9). The cytotoxic activity of the RIPs and the immunotoxins has been measured as either protein synthesis or cell viability inhibition at 24 or 48 h as indicated. The effects of the monoclonal antibody 44G4 and a mixture containing 44G4 and nigrin b are also shown. Data have been obtained from refs. [91,92].

### 5.3. Lectin-Nigrin b Conjugates

SELld is a dimeric D-galactose-binding lectin isolated from the leaves of *Sambucus ebulus* L [50]. Conjugates containing nigrin b as a toxic moiety and the mucin-binding lectin SELld as a carrier molecule, proved to be effective in killing COLO 320 and HeLa cells [118]. 

Conjugation of nigrin b to SELld using SPDP did not affect the intrinsic translational inhibitory activity of the RIP. Instead, it greatly increased the cytotoxicity of nigrin b on COLO 320 cells. The cytotoxic activity of SELd-nigrin b conjugate measured as IC_50_ is close to 100 pM, which is comparable to that of other cytotoxic conjugates and immunotoxins used for experimental cancer therapy [65]. Since the amount of free nigrin b in the conjugate preparation is very low, its contribution to the cytotoxicity of the conjugates is negligible. This indicates that SELld is able to re-direct the conjugate to a more productive pathway than that taken up by nigrin b alone. According to reports indicating that ricin and certain anticancer immunotoxins promote apoptosis [119], nigrin b and the SELld-nigrin b conjugate were found to promote COLO 320 DNA fragmentation at the same concentrations seen to be cytotoxic [118]. 

Other conjugates formed with nigrin b and different lectins such as SNA I, the isolated SNA I B chain, and the SELfd were also evaluated to direct the conjugates to Hela and COLO 320 cells. SNA I is a tetrameric sialic acid-binding lectin from *Sambucus nigra* L. bark composed of two types polypeptide chains A and B [41]. SELfd is a homodimeric lectin from *Sambucus ebulus* L. fruits [30]. These conjugates were constructed in exactly the same way as the SELld-nigrin b conjugate. Only the conjugates made with SELfd displayed an IC_50_ value on COLO 320 cells of 800 pM, which is of the same order of magnitude than that of the SELld-nigrin b conjugate. The conjugates made either with SNA I or the SNA I B chain and nigrin b were less active. At the concentrations used, the free lectins had a very small effect or none on cell viability.

## 6. Conclusions and Perspectives

The results obtained using nigrin b or ebulin l targeted to either CD105 or TfR-expressing tumor cells suggest that these toxins have considerable potential for use in cancer therapy. These studies have shown a clear dependence of the toxin on the transferrin or antiCD105 moiety, suggesting a high selectivity for tumor cells.

A major limitation of immunotoxins is the development of neutralizing antibodies to both the toxic and the carrier portions of the conjugate. The administration of ricin (blocked ricin)-containing immunotoxins to human patients leads to the appearance of neutralizing anti-ricin (HARA) antibodies that neutralize the effectiveness of these immunotoxins with time [78,120]. This immunogenic character is shared by viscumin (ML-1), isolated from the European mistletoe (*Viscum album*). Nigrin b and ebulin l display lower immunogenicity and adjuvanticity in a mouse nasal mucosa model than viscumin [121]. Another problem with the use of ricin immunotoxins is the development of vascular leak syndrome (VLS) as a consequence of damage to epithelial cells in capillaries [122]. The low *in vivo* toxicity of nigrin b and ebulin l makes the appearance of midtime vascular problems such as VLS improbable. In fact, mice treated with a fairly high i.p. dose of nigrin b (10 mg/kg body weight) or ebulin l (1.7 mg/kg body weight) completely recovered at the end of the experiments, at least up to 30 days after toxin administration.

On the other hand, the windows of cytotoxicity between free and conjugated non-toxic type 2 RIPs are crucial for their use as toxic moieties of conjugates and immunotoxins for therapy. Even if the conjugate undergoes complete extracellular hydrolysis, the amount of free nigrin b or ebulin l released would be insufficient to trigger significant toxic effects. This is an enormous advantage as compared with immunotoxins made with other RIPs, such as the ricin A chain purified from the ricin holoenzyme. In that case, even extremely low amounts of ricin B chain contaminant would lead to reconstitution of the highly toxic holoenzyme [1]. 

In conclusion, nigrin b and ebulin l have a number of valuable advantages over ricin and derivatives, the best one probably accounts for their differential cytotoxicity with ricin. Another advantage is bio-safety in the preparation and handling procedures of *Sambucus* RIPs. Future work will investigate large scale preparation of the conjugates with the aim to address their cytotoxicities in a number of potential target cancer cells and its *in vivo* effectiveness.
